# Characterization of the First Carbapenem-Resistant *Pseudocitrobacter faecalis* Harboring *bla*_OXA-181_ in China

**DOI:** 10.3390/antibiotics11060737

**Published:** 2022-05-30

**Authors:** Qingyu Shi, Yan Guo, Yang Yang, Shi Wu, Renru Han, Li Ding, Dandan Yin, Fupin Hu

**Affiliations:** 1 Institute of Antibiotics, Huashan Hospital, Fudan University, Shanghai 200040, China; 21111220064@m.fadan.edu.cn (Q.S.); guoyan@fudan.edu.cn (Y.G.); yangyang_hs@fudan.edu.cn (Y.Y.); wushi68@aliyun.com (S.W.); hanrenru@fudan.edu.cn (R.H.); ding_li@fudan.edu.cn (L.D.); yindandan@fudan.edu.cn (D.Y.); 2 Key Laboratory of Clinical Pharmacology of Antibiotics, Ministry of Health, Shanghai 200040, China

**Keywords:** *Pseudocitrobacter faecalis*, *bla*_OXA-181_, carbapenem, IncX3

## Abstract

With the wide use of carbapenems, carbapenem-resistant *Enterobacterales* have been increasingly reported worldwide. In this study, one *bla*_OXA-181_-positive *Pseudocitrobacter faecalis* strain was isolated from the blood culture of a patient with a bloodstream infection in China, which was its first clinical report outside Pakistan. Species identification of *P. faecalis* was initially performed using MALDI-TOF/MS and further confirmed by 16S rRNA gene and housekeeping gene sequencing. The antimicrobial susceptibility testing was determined through the broth microdilution method, and their clonal relationship was analyzed by pulsed-field gel electrophoresis. To study the transmission and genetic structure of the *bla*_OXA-181_ gene, a transformation test and whole-genome sequencing (WGS) were performed. The results of the antimicrobial susceptibility testing indicated this *P. faecalis* was resistant to carbapenems, quinolones, and commonly used β-lactam/β-lactamase inhibitor combinations. Through WGS and transformation experiments, *bla*_OXA-181_ and *qnrS1* genes causing antibiotic resistance were located on a 55,148-bp length IncX3 type plasmid with a truncated ColKp3 replicon gene. As a rare species of *Enterobacterales*, *P. faecalis* was clinically reported in China for the first time, and the *bla*_OXA-181_ gene it carried was located on a globally disseminated IncX3 plasmid. The spread of such bacteria and antibiotic resistance requires more clinical attention.

## 1. Introduction

The emergence of multidrug-resistant *Enterobacterales* severely threatens public health, and carbapenems have been regarded as its therapeutic choice due to their broad spectrum of activity, stability of extended-spectrum β-lactamase, and proven safety [1,2,3]. However, with rising clinical use, carbapenem-resistant *Enterobacterales* (CRE) have been increasingly reported worldwide. With limited therapeutic regimens, the infections associated with CRE could even lead to a high mortality rate of above 30% [4]. The detection rate of CRE is undergoing rapid growth worldwide, and that in China has increased over 2.5 times during the past fifteen years, and even reached 10.5% in 2021, based on data from the China Antimicrobial Surveillance Network (CHINET) [5,6,7].

The primary resistance mechanism of CRE is carbapenemase production, mainly including Ambler class A (KPC), Ambler class B (NDM, IMP, VIM), and Ambler class D (OXA-48-like) [8]. Worldwide, KPC-type carbapenemase represented the majority, followed by the NDM- and OXA-type [5]. In China, the proportion of OXA-48-like carbapenemase is rising, especially in children, with OXA-232-type as the primary type (97.1%), while in countries such as Angola, OXA-181-type was dominant [9,10]. Worryingly, such resistance was no longer confined to common bacteria, such as *Escherichia coli* and *Klebsiella pneumoniae*, but extensively occurred in other *Enterobacterales* clinical isolates [11].

*Pseudocitrobacter faecalis* was a kind of Gram-negative facultatively anaerobic bacteria discovered in 2010 [12]. It is a rare kind of *Enterobacterales* and has only been clinically reported in Pakistan. Such clinical isolates showed carbapenem resistance due to the production of NDM-1 carbapenemase. Herein, we report the first *bla*_OXA-181_-positive *Pseudocitrobacter faecalis* from a patient with bloodstream infection (BSI) in China.

## 2. Results

### 2.1. Case Presentation

A 19-year-old man was diagnosed with acute myeloid leukemia (AML) type M2 and was first admitted in August 2019. HAA regimen (homoharringtonine, cytarabine, and aclarubicin) was employed as his chemotherapy, while post-chemotherapeutic bone marrow suppression and severe pneumonia caused by bacterial and fungal infection appeared subsequently. On 6 February 2020 (defined as day 1), the patient went through his fifth chemotherapy and soon developed septic shock with positive blood cultures. Meanwhile, lung computed tomography (CT) revealed new ground-glass patchy shadows in the multi-lobar of bilateral lungs. Meropenem (1g q8h) and tigecycline (100g q12h) were used as the empirical therapy based on his previous medical history. Further workup showed that the strains isolated from blood on day 16 were carbapenem-resistant *P. faecalis* strain SC48 and *Klebsiella pneumoniae* strain KPN. Strain KPN was sensitive to major commonly used antibiotics, including cephalosporins, carbapenem, quinolones, etc. Thus meropenem was replaced by ceftazidime (3g q12h) and amikacin (1g QD) 3 days later.

**Figure 1:**
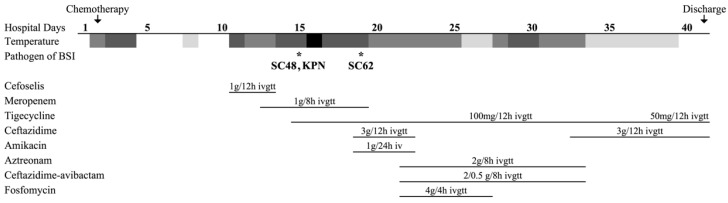
Patient treatment course and microbiological characteristics of patients with bloodstream infection. * Pathogen SC48 and SC62 were *Pseudocitrobacter faecalis* clinical isolates. Pathogen KPN indicated a highly sensitive *Klebsiella pneumoniae* clinical isolate. The blocks from dark to light indicated high, moderate, low-grade fever, and normal temperature.

However, the patient had a recurring fever, and progressing pneumonia was shown by CT reexamination. On day 22, blood culture was positive again with an isolate (strain SC62) similar to strain SC48, so the therapeutic regime was switched to aztreonam, ceftazidime-avibactam, and fosfomycin combined with tigecycline. Mercifully, infection was thereafter brought under control gradually evincing decreased inflammatory index, pulmonary foci absorption, and negative blood culture. For economic concerns, tigecycline and ceftazidime were given again on day 33, based on the clinical situation and the dosage was then reduced by degrees. Antifungal drugs, voriconazole and amphotericin B, covered the whole treatment process. The patient was finally discharged 41 days after admission. The other clinical and microbiologic details are summarized in Figure 1.

### 2.2. Characteristics of P. faecalis Strains

**Figure 2:**
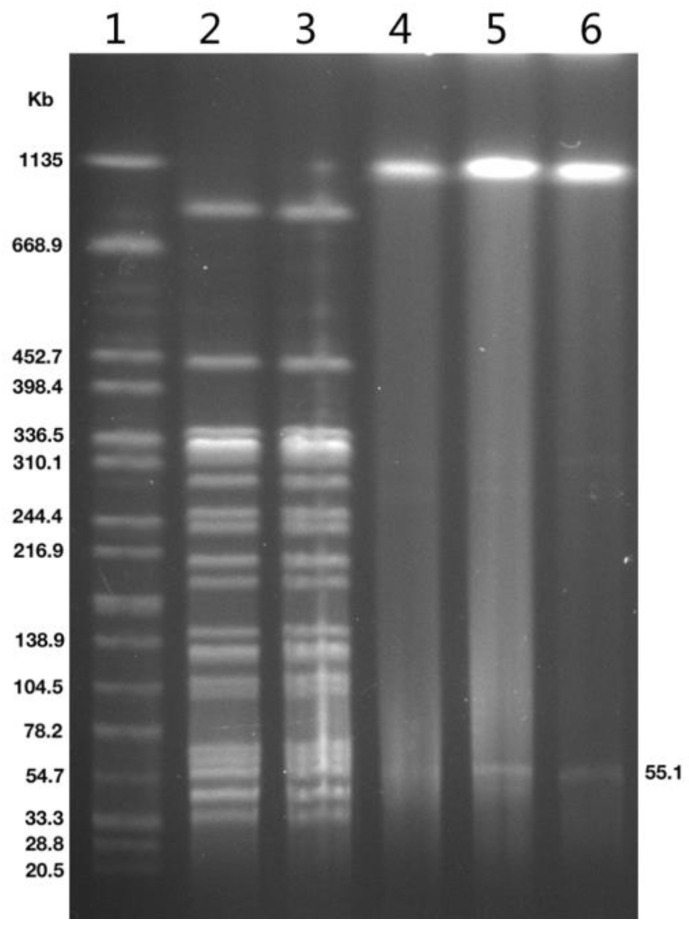
PFGE of *Pseudocitrobacter faecalis* clinical isolates and transformant. Lanes 1, marker *Salmonella braenderup* H9812; line 2 to 3, PFGE image of *P. faecalis* SC48, SC62; line 4 to 6, S1-PFGE image of *P. faecalis* SC48, SC62 and *E. coli* DH5α-SC48-T.

Based on the PFGE fingerprint shown in Figure 2, *P. faecalis* SC48 and SC62 isolated from blood culture were identical in genomic pulsotype. Both isolates were high-level resistant to carbapenems and quinolones (Table 1).

The MICs of the carbapenems ranged from 32 mg/L to 64 mg/L, and that of quinolones ≥ 8 mg/L. Both strains were also resistant to commonly used β-lactam combination agents (cefoperazone-sulbactam and piperacillin-tazobactam) and were intermediate to cefepime. Ceftazidime, aztreonam, ceftazidime-avibactam, trimethoprim-sulfamethoxazole, amikacin, tigecycline, and polymyxin B maintained well in vitro activity for them. The acquisition of *bla*_OXA-181_ carrying by plasmid altered the resistance of transformant *E. coli* DH5α-SC48-T towards β-lactams, increasing the MICs of imipenem, meropenem, cefoperazone-sulbactam, and piperacillin-tazobactam for no less than eight times. Meanwhile, *E. coli* DH5α-SC48-T also acquired resistance to quinolones with an over 4-fold rise in the MICs of ciprofloxacin and levofloxacin (Table 1).

### 2.3. Genetic Analysis of Strains and bla_OXA-181_-Positive Plasmid

**Figure 3:**
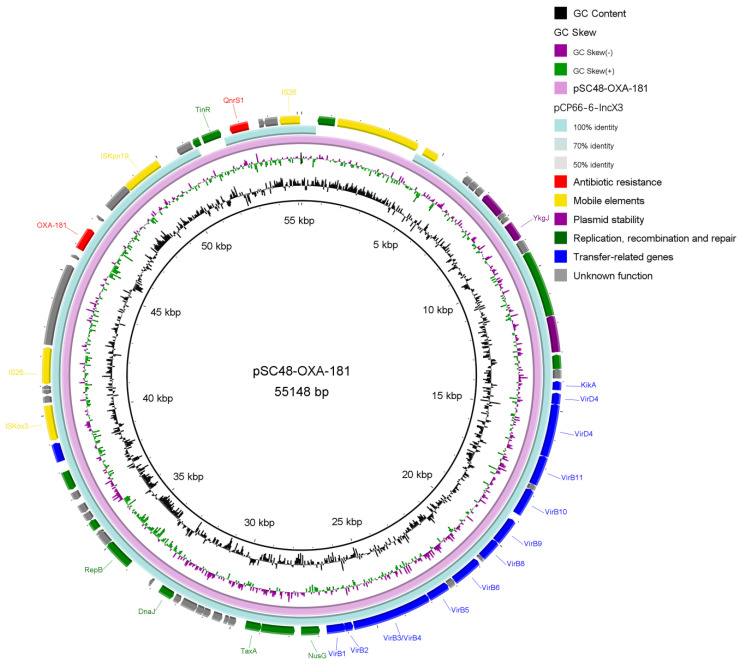
Ring diagram representation of plasmid pSC48-OXA-181. From the inside to the outside: circle 1, scale; circle 2, GC content; circle 3, GC skew; circle 4, ring diagram of pSC48-OXA-181; circle 5, ring diagram of comparative plasmid pCP66-6-IncX3; circle 6, functional classified genes.

Through the WGS analysis, it was found that both *P. faecalis* isolates belonged to the same clinical strain, which contained numerous resistance genes, mainly involving *bla*_DHA-1_, *bla*_OXA-1_, *bla*_OXA-181_, *aac(6’)-IIc*, *aac(6’)-Ib-cr*, *qnrS1*, *qnrB4*, *sul1*, *sul2*, *tet(A)*, *ere(A)*, *mph(A)*, *catB3*, *floR,* and *arr.* Each strain bore two plasmids, one of them was a transmissible plasmid harboring *bla*_OXA-181_ and *qnrS1* that led to carbapenem and quinolone resistance (Figure 2). According to the transformation experiment, the *bla*_OXA-181_ gene was located at a 55,148-bp length IncX3 type plasmid with truncated ColKp3 replicon gene and was named pSC48-OXA-181 (Figure 3).

This plasmid was well-matched with another plasmid previously reported in the same province (pCP66-6-IncX3, GenBank accession no. CP053726.1), showing 91% coverage with perfect identity only with the difference of mobile elements. These mobile elements were distributed nearby resistance gene *bla*_OXA-181_ and *qnrS1* consisting of ISKox3, IS26, and ISKpn19, leading to possible transposon-mediated spread. This plasmid also encoded type IV secretion (T4S) systems mediating transportation, including VirB and VirD proteins.

## 3. Discussion

*Pseudocitrobacter* gen. nov. is a novel genus of the *Enterobacterales* first observed in 2010 with *Pseudocitrobacter faecalis* sp. nov., *Pseudocitrobacter anthropi* sp. nov., and *Pseudocitrobacter vendiensis* sp. nov., among which *P. anthropi* was a later heterotypic synonym of *P. faecalis* [12,13]. Unlike *P. vendiensis,* found only in Denmark and Brazil, *P. faecalis* was sporadically reported in Asia, America, and Africa, mainly from Pakistan, India, China, and America, based on previous reports and data from the GenBank database (https://www.ncbi.nlm.nih.gov/nuccore/?term=%22Pseudocitrobacter+faecalis%22%5Bporgn%3A__txid1398493%5D, (accessed on 15 February 2022)) [12,13,14,15,16,17]. Though *P. faecalis* was only clinically reported in Pakistan, it could be found in the environment, animals, and plants, including foods such as egg, cucumber, and mango.

Herein, *P. faecalis* was first found in China and presumptively associated with bloodstream infection. The prevalence of bloodstream infection increased from 2010 to 2019 in China, among which bacteremia occupied a dominant position (93.1%) [18]. Clinicians attached importance to patients suffering from bloodstream infection, given its high mortality, especially those with high disease severity and inadequate immunologic defenses [19,20]. In this case, neutropenia occurred after high-dose chemotherapy was performed for hematologic malignancy, which significantly impacts the incidence of bloodstream infection in cancer patients [21]. Furthermore, the patient was suggested to have a hospital-acquired bloodstream infection combined with the course of the disease, which was consistent with previous studies showing that the composition ratio of hospital-acquired bloodstream infections was increasing annually and those related to *Enterobacterales* also dynamically increasing [18,22].

The appearance of a *P. faecalis*-related hospital-acquired bloodstream infection suggested the possible emergence and prevalence of such rare bacteria, which gave cause for increased vigilance. It has been proved that international travel can transfer resistant bacteria and antimicrobial resistance genes worldwide [23]. These bacteria and resistance genes are likely to invade travelers and further disseminate in the home country before they are lost in the host [24]. Otherwise, *P. faecalis* has been reported in food and the environment, indicating the possibility that such bacteria and resistance genes have entered the hospital settings via environmental contamination and the food chain [25,26,27].

It is worth noting that all *Pseudocitrobacter* gen. strains mediating clinical infection are associated with resistance genes encoding carbapenemases, including *bla*_NDM-1_, *bla*_KPC-2,_ and *bla*_IMP-1_ [12,13,17]. *P. faecalis* strains producing OXA-181 carbapenemase were isolated in our study. OXA-48-like carbapenemases such as this are generally found in *Enterobacterales* worldwide, which even make up the most prevalent carbapenemase type in countries such as the Netherlands (44%) [28]. In China, OXA-48-like carbapenemase-producers accounted for 7.3% of CRE strains ranking behind KPC-2 and NDM [9]. Though OXA-232-type carbapenemase holds an overall majority, OXA-181 type has successively emerged in *Enterobacterales* since 2014 [29,30].

Globally, *bla*_OXA-181_ was mainly carried by highly conserved plasmids instead of chromosomally localizing. Such plasmids featured the *qnrS1* allele and ColKP3 and IncX3 replicons, which was consistent with our study [31,32,33]. Early research revealed that the *bla*_OXA-181_ was inserted into the IncX3 plasmid through the IS3000-mediated co-integration of the ColKP3-type plasmid [34]. This kind of plasmid was also characterized by Tn3 family transposases and T4S systems mediating DNA transportation, different from those with other *bla*_OXA-48-like_ genes [32,35]. These resistance genes located on plasmids were often colocalized with mobile genetic elements leading to the spread of resistance to carbapenem antibiotics between distinct plasmids and bacteria, which increases the need for vigilance in the clinic [24].

## 4. Materials and Methods

### 4.1. Clinical Isolates and Patient Data

A total of three clinical strains were isolated from blood samples of a patient in a tertiary hospital. Among them, *P. faecalis* SC48 and *P. faecalis* SC62 were carbapenem-resistant, while one *K. pneumoniae* was carbapenem-susceptible. Strain identification was performed by matrix-assisted laser desorption ionization time-of-flight mass spectrometry (MALDI-TOF MS, bioMérieux, Marcy-l’Étoile, France), and further confirmed by PCR of 16S rRNA gene and housekeeping gene sequences [12]. Clinical features of the patient were then systematically obtained through electronic medical records, mainly including age, gender, disease diagnosis and prognosis, specimen origin and date, antibiotics usage, etc.

### 4.2. Antimicrobial Susceptibility Testing

According to the Clinical and Laboratory Standards Institute (CLSI), the minimal inhibition concentration (MIC) was determined through the broth microdilution method [36]. Antimicrobial agents, including imipenem, meropenem, ceftazidime, cefepime, aztreonam, ceftazidime-avibactam, cefoperazone-sulbactam, piperacillin-tazobactam, trimethoprim-sulfamethoxazole, ciprofloxacin, levofloxacin, amikacin, tigecycline, and polymyxin B, were tested and results were interpreted by breakpoints of 2021 CLSI, FDA (for tigecycline only) and EUCAST (for polymyxin B only) [36]. *E. coli* ATCC 25,922 was used as quality control for the antimicrobial susceptibility testing.

### 4.3. Plasmid Transformation Experiments

Plasmid DNAs were extracted from donor *P. faecalis* SC48 by phenol-chloroform method and then electroporated into recipient *E. coli* DH5α. These transformants were selected on Luria–Bertani agar plates containing ampicillin (50 mg/L) and subjected to PCR for detection of the *bla*_OXA-181_ gene using primers OXA-F (5′-GCGTGGTTAAGGATGAACAC-3′) and OXA-R (5′-CATCAAGTTCAACCCAACCG-3′) [37]. All PCR positive products were sequenced and comparatively analyzed for homology using BLASTn algorithms (http://blast.ncbi.nlm.nih.gov/Blast.cgi, (accessed on 15 February 2022)).

### 4.4. Pulsed-Field Gel Electrophoresis (PFGE)

With *Salmonella braenderup* H9812 as the reference marker, the clonality and plasmids of strains were confirmed by PFGE and S1-PFGE [38]. Briefly, bacterial DNA of *P. faecalis* was digested with the restriction endonuclease *XbaI* and that of the donor strain and transformant with S1-nuclease (TaKaRa, Beijing, China). PFGE was carried out at 14 °C for 20 h using a CHEF Mapper system (Bio-Rad Laboratories, Hercules, CA, USA).

### 4.5. Whole-Genome Sequencing and Analysis

Total DNA of *P. faecalis* strain SC48, SC62, and transformant *E. coli* DH5α-SC48-T was extracted and subjected to whole genome sequencing (WGS) via Illumina paired-end sequencing (Illumina, San Diego, CA, USA), and then de novo assembled by SPAdes 3.12.0 [39]. Antimicrobial resistance genes were analyzed by ResFinder 4.1 (https://cge.food.dtu.dk/services/ResFinder/, (accessed on 15 February 2022)) with a 90% threshold for gene identification and a 60% minimum length coverage.

## 5. Conclusions

As a rare species of *Enterobacterales*, a *P. faecalis*-mediating fatal bloodstream infection was found in China, the first to be clinically reported outside Pakistan since its discovery. The *bla*_OXA-181_ gene carried by *P.* *faecalis* was located on a globally disseminated IncX3 plasmid. The *P. faecalis*-related nosocomial infection indicated the potential worldwide spread of such bacteria, which was considered to be rare. Population mobility and environmental pollution might be the reason, so more clinical attention is required.

## Figures and Tables

**Table 1:** Minimal inhibitory concentrations (MICs) of *Pseudocitrobacter faecalis* clinical isolate, transformant, and recipient.

Strains	β-Lactamase Genes	Fluoroquinolone- Resistant Genes	MIC (mg/L) ^a^													
			IMP	MEM	CAZ	FEP	ATM	CZA	SCF	TZP	SXT	CIP	LEV	AMK	TGC	POL
*P. faecalis* SC48	*bla*_DHA-1_, *bla*_OXA-1_, *bla*_OXA-181_	*qnrB4*, *qnrS1*, *aac(6’)-Ib-cr*	64	32	2	8	≤1	1	128	>256	0.5	>8	8	4	1	0.5
*P. faecalis* SC62	*bla*_DHA-1_, *bla*_OXA-1_, *bla*_OXA-181_	*qnrB4*, *qnrS1*, *aac(6’)-Ib-cr*	64	64	2	8	≤1	1	128	>256	0.5	>8	8	8	1	0.5
*E. coli* DH5α- SC48-T	*bla*_OXA-181_	*qnrS1*	1	0.25	0.5	0.125	≤1	0.25	8	64	≤0.25	0.25	0.5	≤1	0.25	0.25
*E. coli* DH5α	-	-	0.125	≤0.03	0.5	≤0.06	≤1	0.125	≤1	4	≤0.25	≤0.06	≤0.125	≤1	0.125	0.25

^a^ IPM, imipenem; MEM, meropenem; CAZ, ceftazidime; FEP, cefepime; ATM, aztreonam; CZA, ceftazidime-avibactam; SCF, cefoperazone-sulbactam; TZP, piperacillin-tazobactam; SXT, trimethoprim-sulfamethoxazole; CIP, ciprofloxacin; LEV, levofloxacin; AMK, amikacin; TGC, tigecycline; POL, polymyxin B.

## Data Availability

The nucleotide sequence of pSC48-OXA-181 containing *bla*_OXA-181_ and *qnrS1* was deposited in the GenBank under accession number OK558605.
